# Yellow Fever Outbreaks and Reactive Vaccination in Africa, 2010–2025: A Scoping Review and Analysis

**DOI:** 10.21203/rs.3.rs-10538163/v1

**Published:** 2026-07-31

**Authors:** Carlos Haring, Sayana Lee, David Dowdy, Seth Judson

**Affiliations:** Johns Hopkins University; Scripps College; Johns Hopkins University; University of California, Los Angeles

**Keywords:** Yellow fever, outbreaks, vaccination, mortality, Africa

## Abstract

Containing yellow fever (YF) outbreaks rapidly through reactive vaccination campaigns (RVCs) is a key objective of the 2017–2026 Eliminate Yellow Fever Epidemics (EYE) strategy. We conducted a scoping review and quantitative analysis using grey and peer-reviewed literature on YF outbreaks in Africa (2010–2025) to compare how factors including outbreak size and timing of RVCs have changed pre- and post-EYE strategy. The overall median time between YF index case and RVC was 90.5 days (interquartile range [IQR]: 53.8–113.2), with medians of 108 days (IQR 59–111) for 2010–2016 and 79 days (IQR 51–114.5) for 2017–2025. The median (IQR) reported cases per outbreak was 91 (IQR: 16–276) in 2010–2016 and 94 (49–377) in 2017–2025, with case fatality ratios (CFRs) of 8.5% (standard error [SE]: 2.1%) and 4.9% (SE: 2.9%), respectively. High morbidity and mortality for YF persist in Africa, and more timely RVCs will be required to achieve YF control.

## Introduction

Yellow fever (YF) is a viral hemorrhagic fever caused by the YF virus, which is transmitted by mosquitoes in Africa and South America. In December 2015, a YF outbreak that started in Angola and spread to the Democratic Republic of the Congo (DRC) depleted the global supply of YF vaccines.^1^ In response, the Eliminate Yellow Fever Epidemics (EYE) strategy was implemented in 2017, with three main objectives: “protect at-risk populations,” “prevent international spread,” and “contain outbreaks rapidly.”^2^ Increasing baseline YF immunization coverage and decreasing time to reactive vaccination during outbreaks are key components to achieving these objectives.

Yellow fever immunization coverage in at-risk areas has increased greatly since the EYE strategy with an estimated 336 million individuals vaccinated in Africa since 2017.^3^ The transition of the EYE strategy in 2026, however, raises key questions regarding the future of YF prevention in Africa.^4^ Evaluating the current state of YF outbreaks and response is therefore crucial as transition plans are made, both globally and at country-level. Modeling studies have shown that vaccination campaigns have decreased YF outbreaks and disease burden,^5–8^ while vaccination delays can increase outbreak size.^9^ However, it is less well understood how YF outbreak patterns and responses have changed since EYE strategy implementation. With the infusion of support from the EYE strategy, it is presumed that the time from YF outbreak detection to reactive vaccination has shortened since 2016, but the extent of this improvement and any corresponding changes in outbreak size/severity remain unknown. Therefore, the objectives of our scoping review were to 1) describe the size and severity of YF outbreaks; 2) describe the timing and rapidity of reactive vaccination campaigns (RVCs); and 3) compare these indicators in the pre-EYE (2010–2016) and post-EYE (2017–2025) periods in Africa.

## Methods

### Literature Review of Yellow Fever Outbreaks

We conducted a scoping review, including both publications and grey literature in English, of YF outbreaks from 2010–2025 using the Preferred Reporting Items for Systematic reviews and Meta-Analyses extension for Scoping Reviews (PRISMA-ScR) guidelines (Appendix **Figure S1, Table S1, Table S2**). If conflicting information was present, data from the most recent document or peer-reviewed paper was used along with epidemiological context. Two reviewers (CH and SL) checked the validity of the outbreak profiles and the data entries for each outbreak with the corresponding documents.

Yellow fever outbreaks were classified as events with at least one confirmed YF case using the World Health Organization (WHO) definition.^10^ Furthermore, to avoid double counting, when an RVC was initiated in response to multiple separately-reported YF events, those events were grouped together as a single outbreak. The index case date was defined as the date when the first suspected YF case reported by health authorities presented to clinicians or to an investigative team. The date of symptom onset was used if the presentation date was unavailable. Outbreaks where the epidemiological link between index case and reactive vaccination was uncertain, or where response times were unusually long during the COVID-19 pandemic, were excluded from the time-to-RVC analysis. RVC coverage was defined as the proportion of the estimated target population that was vaccinated by during the RVC. The vaccination rate was defined as the percentage of the target population vaccinated per day. The calculations for the vaccination coverage and rates are in the Appendix.

Descriptive statistics were calculated for these outbreaks using R (version 4.5.1). We compared results before and after the 2016 Angola/DRC outbreak that led to the EYE strategy in 2017. To compare changes between these periods, we used a cutoff date of February 14, 2017, when the DRC outbreak was declared over.^11^ No new outbreaks were identified between January 1, 2017, and February 14, 2017, so we refer to these periods as “2010–2016” and “2017–2025”, respectively. The Wilcoxon rank sum test was used to compare continuous, non-normally distributed variables (time from index/outbreak confirmation to first reactive vaccination, number of reported cases, and RVC coverage). Welch’s t-test was used for pooled estimates of continuous variables (reactive vaccination rates and case fatality ratio [CFR]). The calculation of vaccination rates, CFR, and the corresponding variances are further described in the Appendix, along with a table describing all RVCs (**Table S3**).

## Results

### Time to reactive vaccination

We obtained data on 43 total YF outbreaks with RVCs in Africa between 2010–2025, of which 26 (60%) occurred in 2017–2025. After removing outliers, the dates of YF index cases and RVCs were confirmed for 20 outbreaks (11 in 2017–2025) (Table 1). The shortest interval from index case presentation to reactive vaccination occurred during the 2021 Ghana outbreak (17 days), while the longest delay occurred during the 2022 Kenya outbreak (192 days). The overall median time from the index case to reactive vaccination was 90.5 days (interquartile range [IQR]: 53.8–113.2, range 17–192) (Table 1). This delay was only modestly shorter when comparing outbreaks occurring in 2010–2016 (median 108 days, IQR: 59–111) versus 2017–2025 (median 79 days, IQR: 51–114.5, p = 0.55 for difference).

### Reactive vaccination coverage and rate

There were 29 YF RVCs identified with complete data; 11 (38%) occurred prior to the end of the 2016 Angola/DRC outbreak and 18 (62%) after. The overall pooled vaccination rate across the 2010–2025 period was 7.9% (standard error [SE]: 2.0%) of the target population vaccinated per day. Since the end of the 2016 Angola/DRC outbreak, the pooled vaccination rate increased from 6.1% (SE: 3.0%) to 9.8% (SE: 1.0%) of the target population per day, though this was not statistically significant (p = 0.27). The median coverage for YF RVCs during the 2010–2025 period was 94% of the target population (IQR: 88%−102%).

### Morbidity and mortality

**Figure 2:**
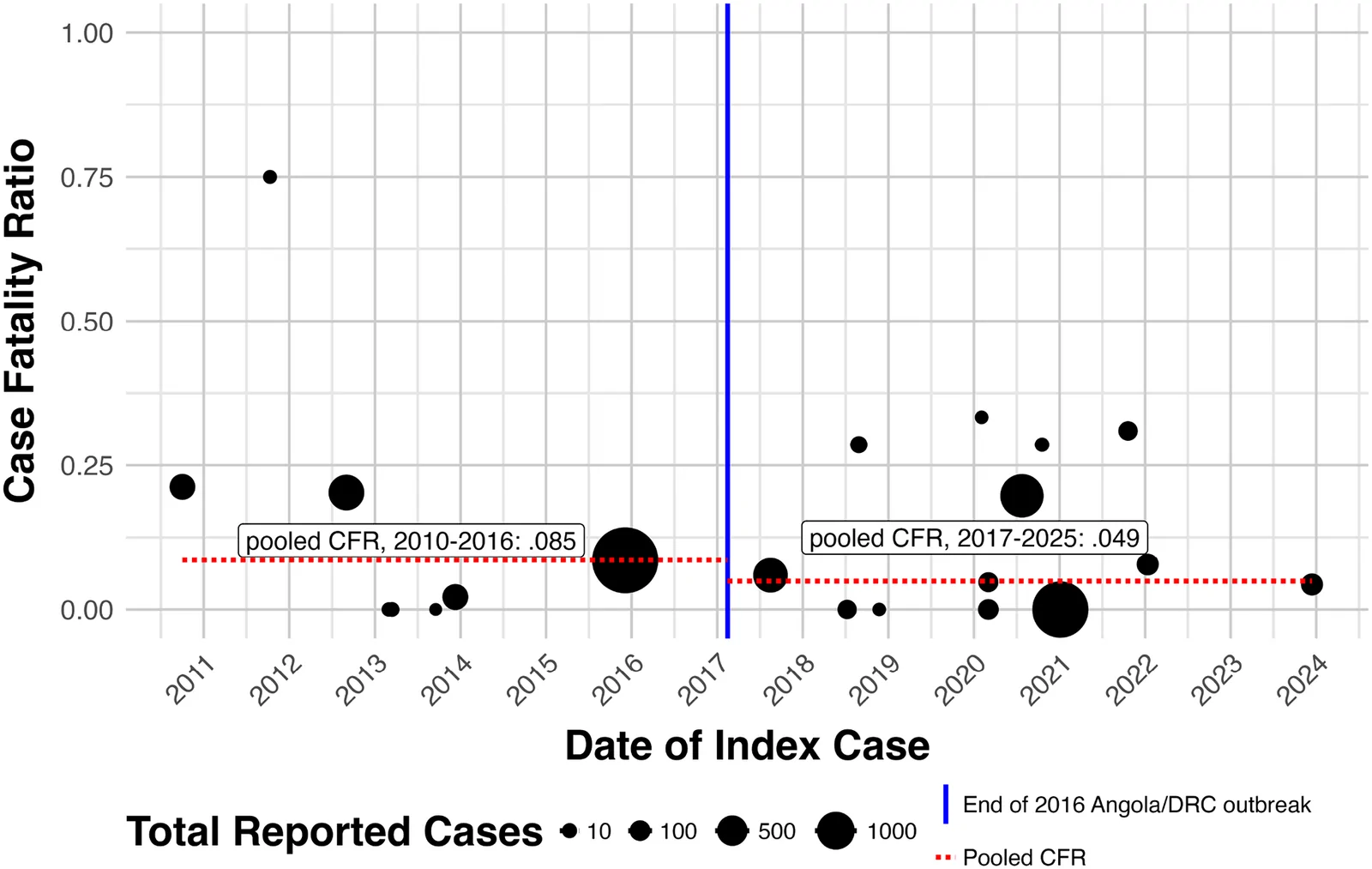
Case fatality ratio (CFR) of yellow fever outbreaks in Africa, 2010–2025. Each dot represents the date of an index case onset or presentation for a yellow fever (YF) outbreak in Africa between 2010–2025. The size of each dot represents the number of reported cases in the outbreak while the position indicates the outbreak's case fatality ratio (CFR). The vertical blue line indicates the end of the 2016 Angola/Democratic Republic of the Congo (DRC) outbreak. The dashed red line represents the pooled CFR for each period.

There were 35 YF outbreaks with complete case and mortality data identified; 15 (43%) occurred prior to the end of the 2016 Angola/DRC outbreak and 20 (57%) occurred after. The overall median size YF outbreaks was 91 reported cases (IQR: 30–276). The largest outbreak was in Angola/DRC 2016 (4,618 reported cases in Angola and 2987 in the DRC), and the smallest outbreak was in Cameroon 2013–2014 (2 reported cases). The median outbreak size was 91 cases (IQR: 16–276) in 2010–2016, compared to 94 cases (IQR: 49–377) in 2017–2025 (p = 0.77). The overall CFR among YF outbreaks was 6.6% (SE: 2.0%): 8.5% (SE: 2.1%) in 2010–2016 and 4.9% (SE: 2.9%) in 2017–2025 (p = 0.32) (Figure 2).

## Discussion

This scoping review and analysis provides several insights into YF outbreaks and RVCs in Africa. Although the timing from index case to RVC has fallen slightly after EYE strategy implementation, this decline has been modest (from 108 to 79 days) and not statistically significant. Importantly, substantial delays still exist, with five of the eight most recent outbreaks reporting delays of over 100 days. These delays could reflect gaps in YF diagnostic networks, for example delays between YF specimen collection and testing.^12,13^ Alternatively, there could be limited in-country YF vaccine supply, delays in procuring additional vaccines, or delays in RVC implementation. Understanding which steps are hindering timely reactive vaccination is key for improving future outbreak control as the EYE strategy transitions at the end of 2026.

In addition to variability in outbreak response timing, there was also heterogeneity in the rate and coverage of RVCs – with similar modest (and non-statistically significant) improvements following EYE strategy implementation. Of note, RVC coverage estimates of the target population, such as those over 100%, may represent inaccuracies in population estimates and intra-region mobility.^3,14^ Further evaluation of outbreaks with more robust vaccination rates could provide insight to inform more effective RVCs in the future.

Unfortunately, large YF outbreaks with high case fatality continue to be reported since the launch of the EYE strategy. These outbreaks may reflect the changing epidemiology of YF rather than any failure of the EYE strategy. In addition to YF outbreaks re-occurring in regions considered high-risk (such as West and Central Africa)^15^, there have been outbreaks in new regions, such as East Africa (South Sudan, Kenya, and Ethiopia). Therefore, expanded YF vaccination coverage may be needed in areas previously considered low-risk. The persistently high CFR likely represents the ongoing lack of available therapeutic options for YF.

Comparisons of outbreak size, response, and CFRs are limited by variability in surveillance systems and case reporting biases. The EYE strategy focus on increasing diagnostic capacity could contribute to more cases being detected since 2017 – including more mild cases, lowering the CFR. Furthermore, not every source may have defined cases in the same manner. Other limitations to this analysis include confounding eco-epidemiological factors that could affect outbreak size and vaccination timing. These results should be interpreted as descriptive in nature, rather than an assessment of the causal impact of the EYE strategy.

Yellow fever remains an ongoing public health threat, and large outbreaks continue to occur – many with high CFRs and delays of 100 days or more to the initiation of RVCs. Such high mortality and persistent delays in outbreak responses illustrate the importance of continuing to prioritize a robust YF response, particularly given the 2026 transition of the EYE strategy. Increasing baseline vaccination coverage, decreasing the time to reactive vaccination, and developing YF therapeutics are all essential to achieving the goal of minimizing the burden of this preventable infectious disease.

## Supplementary Files

This is a list of supplementary files associated with this preprint. Click to download.

- Appendix.docx

## Figures and Tables

**Figure 1:**
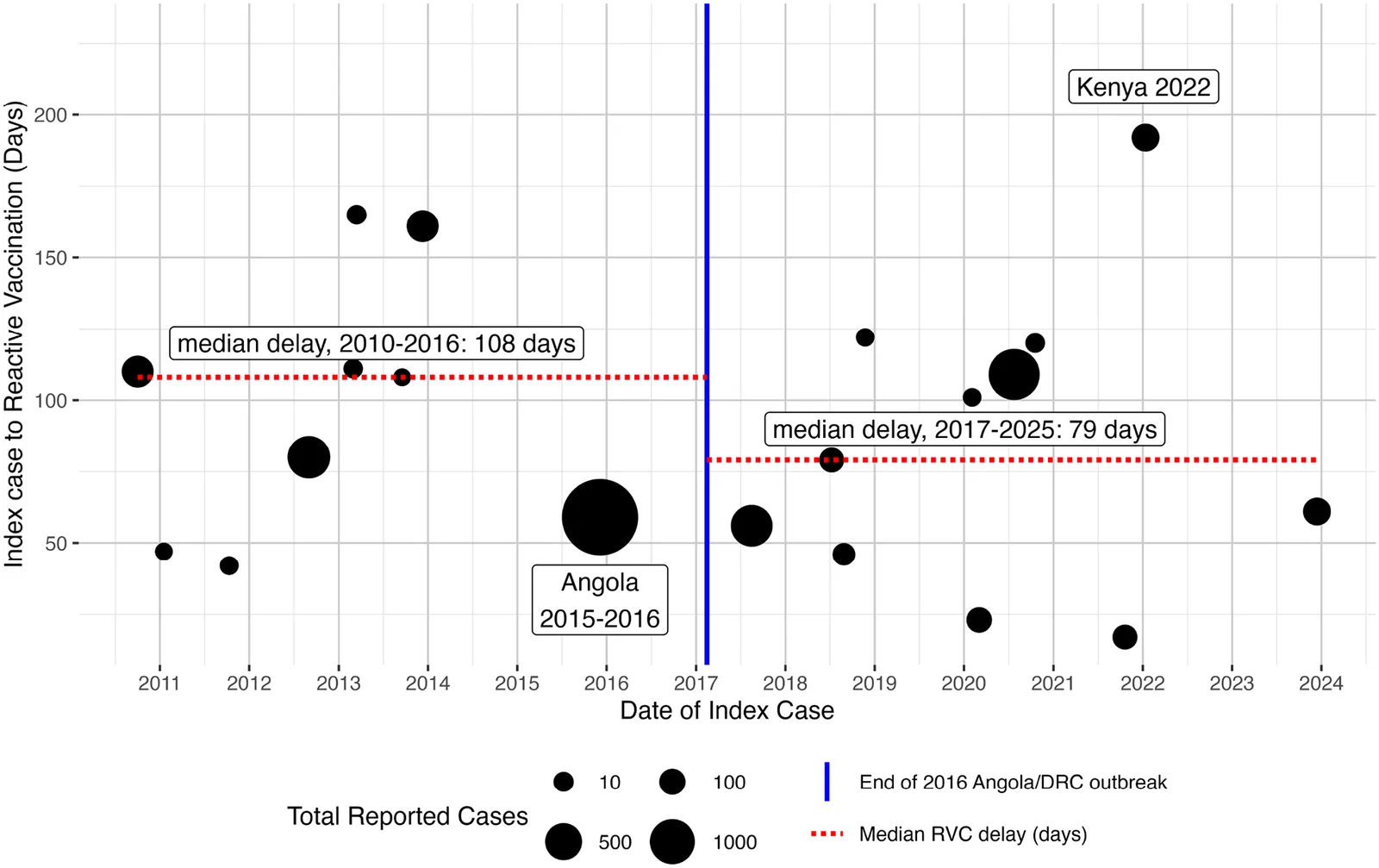
Timing of index case to reactive vaccination for yellow fever in Africa, 2010–2025. Each dot represents the date of an index case onset or presentation for a yellow fever (YF) outbreak in Africa between 2010–2025. The size of each dot represents the number of reported cases in the outbreak while the vertical position indicates the time from index onset or presentation to the first YF reactive vaccination campaign (RVC). The vertical blue line indicates the end of the 2016 Angola/Democratic Republic of the Congo (DRC) outbreak of YF. The dashed red line represents the median RVC delay for each period. Outliers in time to RVC and total reported cases are labeled.

**Table 1.** Yellow fever outbreaks in Africa (2010–2025) included in the time of reactive vaccination analysis.

Year	Country	Days: Index Case to Reactive Vaccination	Total Cases	Total Deaths	CFR^a^
2010	Uganda	110	273	58	0.21
2011	Sierra Leone	47	2	Unknown	Unknown
2011	Ghana	42	4	3	0.75
2012	Sudan	80	844	171	0.2
2013	DRC^b^	111	6	0	0
2013	Cameroon, Littoral	165	8	0	0
2013	Cameroon, Western/Central	108	2	0	0
2013	DRC	161	278	6	0.02
2015	Angola	59	4618	394	0.09
2017	Nigeria	56	778	46	0.06
2018	Congo	79	70	0	0
2018	Ethiopia	46	35	10	0.29
2018	South Sudan	122	3	0	0
2020	Togo	101	3	1	0.33
2020	Ethiopia	23	85	4	0.05
2020	Nigeria	109	1502	296	0.2
2020	Senegal	120	7	2	0.29
2021	Ghana	17	71	22	0.31
2022	Kenya	192	141	11	0.08
2023	South Sudan	61	139	6	0.04

Days: median = 90.5, IQR = 53.8–113.2; range = 17.0–192.0; n = 20 complete outbreaks

a. Case Fatality Ratio, equivalent to the number of total deaths divided by the number of total cases

b. Democratic Republic of the Congo

Sources for outbreak information are in Table S3 with the corresponding reactive vaccinations

## Data Availability

All of the data and R scripts from this study are available at: https://github.com/CarlosZHaring/Yellow-Fever-Outbreaks-and-Reactive-Vaccination-in-Africa-2010-2025-A-Scoping-Review-and-Analysis.git
